# Spinal Cord Stimulation in Painful Diabetic Neuropathy: Advances, Outcomes, and Future Directions

**DOI:** 10.3390/biomedicines13123063

**Published:** 2025-12-12

**Authors:** Roberto Gazzeri, Jacopo Mosca, Felice Occhigrossi, Marcelo Galarza, Riccardo Schiaffini, Giustino Varrassi, Marco Mercieri, Matteo Luigi Giuseppe Leoni

**Affiliations:** 1 Interventional and Surgical Pain Management Unit, San Giovanni-Addolorata Hospital, 00184 Rome, Italy; 2 Anesthesiology, Critical Care and Pain Medicine, La Sapienza University of Rome, 00185 Rome, Italy; 3 Regional Service of Neurosurgery, “Virgen de la Arrixaca” University Hospital, 30001 Murcia, Spain; 4 Endocrinology and Diabetes Unit, Bambino Gesù Children’s Hospital, IRCCS, 00163 Rome, Italy; 5 Fondazione Paolo Procacci, 00193 Rome, Italy; 6 Department of Medical and Surgical Sciences and Translational Medicine, Sapienza University of Rome, 00135 Rome, Italy

**Keywords:** painful diabetic neuropathy, diabetes, spinal cord stimulation, neuropathic pain, neuromodulation, high-frequency stimulation, differential target multiplexed stimulation

## Abstract

Painful diabetic neuropathy (PDN) is a common complication of diabetes mellitus, often inadequately managed by conventional pharmacological therapies. Alternative interventions, including spinal cord stimulation (SCS), have garnered increasing attention for their potential effectiveness. This narrative review evaluates the efficacy, safety, mechanisms of action, and impact on quality of life of SCS in the management of PDN. A systematic search was conducted covering the last 15 years (from January 2010 to April 2025), using the keywords “diabetic neuropathy,” “spinal cord stimulation,” “neuropathic pain,” and “neuromodulation” with Boolean operators. Eligible studies included randomized controlled trials (RCTs), observational studies, systematic reviews, and meta-analyses involving adult populations and published in English. Study selection and data extraction were performed independently by two authors. Multiple RCTs and observational studies (involving over 500 patients) were identified. RCTs consistently demonstrated that SCS significantly reduces neuropathic pain intensity compared to conventional medical therapy (CMT). The most recent study, with the largest sample size (216 patients), reported that high-frequency SCS achieved ≥50% pain relief in 79% of patients at 6 months, compared to only 5% in the CMT group. Observational studies confirmed sustained pain relief (≥50% reduction) in approximately 55–80% of patients over 5–10 years. Significant improvements in sleep, neurological function, and quality of life were also consistently observed. The complication rate was low, with infections requiring explantation in approximately 2–3% of cases. Rare serious adverse events, such as spinal hematoma, were reported. Current evidence underlines the efficacy and safety of SCS, particularly newer waveform paradigms, for the treatment of PDN refractory to medical management. Given its durable effects on pain relief, functional improvement, and quality of life, SCS should be considered an option within the treatment algorithm for carefully selected patients with severe, refractory PDN.

## 1. Introduction

Diabetic neuropathy (DN) is one of the most common and clinically significant chronic microvascular complications of diabetes mellitus, affecting up to 50% of diabetic patients after 10–20 years of disease progression [1]. The most frequent clinical manifestation is symmetric sensorimotor peripheral polyneuropathy, characterized by chronic neuropathic pain. This pain is typically described as burning, tingling, electric shock-like sensations, and hyperalgesia, often distributed in a classic “stocking-glove” pattern involving both lower and upper limbs [2]. From a pathophysiological perspective, DN arises from a complex interplay of mechanisms, including metabolic abnormalities (e.g., chronic hyperglycemia, oxidative stress), microvascular ischemia, direct neuronal injury, neuroinflammation, and progressive nerve fiber degeneration [3]. These processes lead to functional impairment of sensory, motor, and autonomic fibers, resulting not only in neuropathic pain but also in significant disability and functional decline [4]. The clinical burden of painful diabetic neuropathy (PDN) is substantial, as it significantly impairs the quality of life through reduced mobility, sleep disturbances, mood disorders, and decreased independence, ultimately contributing to a greater socio-economic and healthcare burden [5]. The standard therapeutic approach for PDN primarily relies on pharmacological interventions. International guidelines recommend anticonvulsant agents (pregabalin, gabapentin), tricyclic antidepressants (amitriptyline, nortriptyline), and serotonin-norepinephrine reuptake inhibitors (duloxetine, venlafaxine) [6]. Pregabalin has an estimated number needed to treat (NNT) of approximately 6.3 for achieving at least 50% pain reduction, with a number needed to harm (NNH) of about 7.4 for adverse events leading to discontinuation [7]. Gabapentin demonstrates a similar NNT of 6 (95% CI: 4.6–8.3), with NNHs ranging from 8 for dizziness to 21 for peripheral edema [8]. Duloxetine shows an NNT of around 5 (4.9–5.2) and an NNH of 17 for adverse events leading to discontinuation [9]. Amitriptyline is notably effective, with a low NNT of 2 (95% CI: 1.7–2.5), although it is associated with a relatively low NNH of 6 for minor side effects such as dry mouth and somnolence, and 28 for major adverse events leading to treatment discontinuation [10]. Additionally, weak or strong opioid analgesics may be considered in selected cases, despite significant risks of tolerance, dependence, and long-term adverse effects [11]. However, the clinical efficacy of these therapeutic strategies is often limited: approximately 40–50% of treated patients report only partial or unsatisfactory pain relief, frequently accompanied by disabling adverse effects such as somnolence, dizziness, cognitive impairment, and gastrointestinal disturbances [12]. Moreover, many patients require complex combination regimens to achieve adequate pain control [13]. Nutraceuticals represent a promising complementary option in the management of PDN, owing to their antioxidant, anti-inflammatory, and neuroprotective properties. Alpha-lipoic acid (ALA), a potent antioxidant, has demonstrated significant reductions in neuropathic pain and improvements in nerve conduction, although further studies are needed to confirm its long-term efficacy [14]. Benfotiamine, a lipid-soluble derivative of vitamin B1, has shown improvement in sensory symptoms and nerve conduction parameters due to its high bioavailability [15]. Acetyl-L-carnitine (ALC) exhibits neuroprotective effects, with meta-analyses supporting its efficacy in reducing neuropathic pain and improving nerve function [16]. Vitamin D supplementation has been associated with improvements in neuropathy symptoms and quality of life among diabetic patients, though optimal dosage and treatment duration remain to be defined [17]. Gamma-linolenic acid (GLA), found in evening primrose and borage oil, possesses anti-inflammatory properties, though clinical evidence for its efficacy in PDN remains limited and inconsistent [18]. Palmitoylethanolamide (PEA), an endogenous analgesic and anti-inflammatory agent, has shown significant benefit in neuropathic pain relief with a favorable safety profile [19]. Curcumin, derived from turmeric, has recognized antioxidant and anti-inflammatory properties; however, its clinical use is limited by poor bioavailability, necessitating the development of enhanced formulations [20]. B vitamins (B1, B6, B12) are essential for nerve function, and supplementation may alleviate PDN symptoms—particularly in deficient patients—though evidence of general efficacy remains variable [21]. Further high-quality studies are required to establish the long-term safety and effectiveness of nutraceuticals in PDN management. Lastly, cannabis-based medicines may offer potential benefits in alleviating pain and other symptoms associated with peripheral neuropathy; however, further high-quality studies are needed to clarify their efficacy, safety, optimal dosing, and long-term outcomes [22].

Given the limitations of current pharmacological approaches, recent years have seen growing interest in alternative interventional strategies, particularly neuromodulation techniques such as spinal cord stimulation (SCS). This method, which involves the implantation of epidural electrodes, enables targeted modulation of central nociceptive pathways. It has shown potential for significant improvements in refractory neuropathic pain, functional status, and overall quality of life in patients with PDN unresponsive to conventional treatments.

## 2. Spinal Cord Stimulation and Painful Diabetic Neuropathy: Study Selection

A narrative review was conducted to identify and evaluate relevant studies on the use of SCS for the management of PDN, published between January 2010 and April 2025. The electronic databases PubMed, Scopus, and Web of Science were systematically searched. The search strategy was developed using a combination of Medical Subject Headings (MeSH) and free-text terms, targeting the keywords “diabetic neuropathy,” “painful diabetic neuropathy,” “spinal cord stimulation,” “neuropathic pain,” and “neuromodulation.” The review included English studies, involving adult human participants, and reporting on clinical outcomes or safety of SCS for PDN. Additional articles were identified through manual searches of bibliographies from relevant reviews and clinical trials. All duplicates were removed. Titles and abstracts were independently screened by two reviewers (RG and JM), with any discrepancies resolved by a third author (MLGL). The research targeted studies exclusively involving adult patients (aged ≥ 18 years) diagnosed with PDN. Eligible publications included randomized controlled trials (RCTs), prospective observational studies, and retrospective analyses, provided they were published in English and evaluated the efficacy, safety, mechanisms of action, or impact on quality of life of SCS as a therapeutic intervention. Exclusion criteria comprised case reports, case series, editorials, letters, non-peer-reviewed articles, as well as systematic reviews and meta-analyses. Studies on pediatric populations (patients <18 years), those written in languages other than English, and studies not specifically focused on PDN treated with SCS were also excluded. We included studies spanning from 2014 to present to provide both historical context and a complete overview of the available clinical evidence on SCS for PDN. Given the limited number of published trials in this field, older studies remain relevant for understanding the evolution of treatment approaches and identifying persistent methodological challenges.

**Figure 1:**
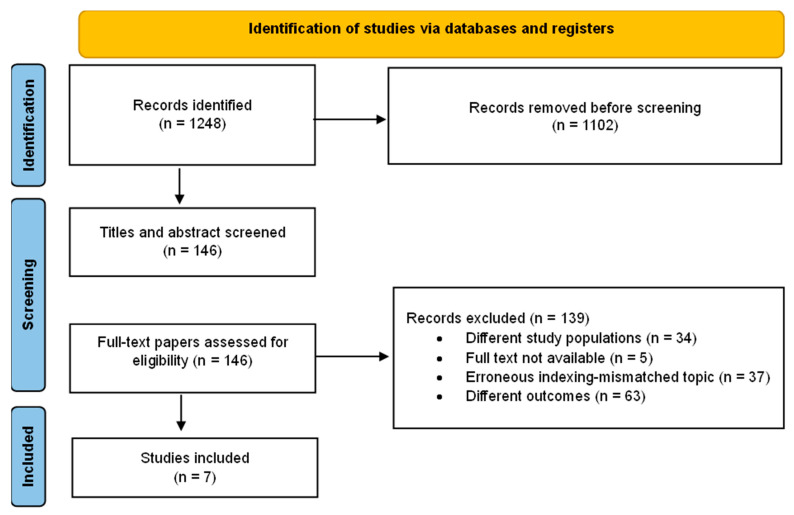
Flowchart of study selection process for SCS research.

A total of seven studies [23,24,25,26,27,28] involving 538 patients were included in this review (Figure 1).

These articles were analyzed to assess clinical effectiveness, safety profile, quality of life outcomes, and potential mechanisms of action associated with SCS for the treatment of PDN. All participants were adults with a diagnosis of long-standing type 1 or type 2 diabetes, presenting with chronic, bilateral neuropathic pain predominantly affecting the lower limbs. In most trials, the duration of neuropathic pain prior to SCS intervention exceeded 12 months, and all patients had failed to achieve satisfactory pain relief despite optimized pharmacological therapy, including gabapentinoids, tricyclic antidepressants, serotonin-norepinephrine reuptake inhibitors (SNRIs), and opioids. Inclusion criteria for RCTs commonly required a baseline pain intensity of 40–50 mm or more on the Visual Analog Scale (VAS) and documented refractoriness to guideline-recommended medications. In some long-term studies, neuropathy severity was further quantified using standardized clinical instruments such as the Michigan Diabetic Neuropathy Score (MDNS), with higher scores often associated with less favorable therapeutic outcomes. Most patients exhibited moderate to severe sensory loss and were receiving polypharmacy at baseline. Notably, in a study including patients with comorbid conditions such as ischemic diabetic foot [27], additional complicating factors such as peripheral vascular disease and tissue hypoxia were present, further exacerbating the complexity of the neuropathic pain presentation. Stimulation parameters varied considerably across the seven studies. Three studies did not report stimulation parameters [23,24,28]. Among studies that reported parameters, frequencies ranged from 30 Hz to 10 kHz, pulse widths from 150 μs to 450 μs, and amplitudes from 0.5 to 3.5 mA. This heterogeneity in stimulation protocols, combined with different primary outcomes and follow-up timepoints (ranging from 6 months to 8–10 years), limits direct comparisons between studies and highlights the need for standardized SCS protocols in diabetic neuropathy treatment. Table 1 provides a summary of the key clinical studies evaluating the efficacy, safety, and long-term outcomes of SCS in patients with PDN, highlighting variations in study design, sample size, and primary endpoints across different research settings.

A detailed summary of outcomes from the SENZA-PDN randomized program and its extensions (6, 12, 24 months, and ~4 years) is provided in Table 2.

## 3. Evidence of Clinical Efficacy

### 3.1. Pain Intensity Reduction (VAS/NRS)—Subjective Outcomes at Short-, Medium-, and Long-Term Follow-Up

Reduction in pain intensity was the primary outcome in all included studies and was most commonly assessed using the VAS or the Numeric Rating Scale (NRS). A reduction of ≥50% in baseline pain was generally considered the threshold for clinically meaningful treatment success. At 6 months, the studies by de Vos et al. (2014) [23], Slangen et al. (2014) [24], Duarte et al. (2016) [28], and Petersen et al. (2021) [25] reported significant pain relief following SCS. Notably, the SENZA-PDN trial demonstrated a substantial reduction in VAS scores, from 7.6 cm at baseline to 1.7 cm in the SCS combined with conventional medical management (CMM) group, with no significant change in the control group receiving CMM alone [38]. Duarte et al. [28] similarly observed greater reductions in pain scores in the SCS group compared to patients receiving conventional medical practice (CMP) after 6 months. At 24 months, Petersen et al. [25] confirmed the sustained efficacy of SCS, reporting a mean 79.9% reduction in pain from baseline. Moreover, 90.1% of patients continued to experience at least a 50% reduction in pain, underscoring the durability of the analgesic effect over the medium term. In a five-year follow-up study, Yi Liu et al. (2025) [39] found that 77% of patients maintained clinically significant pain relief, further supporting the long-term effectiveness and sustainability of high-frequency (10 kHz) SCS therapy for PDN. Beyond randomized evidence, a recent prospective single-center cohort [29] including patients with painful polyneuropathy from multiple etiologies (notably including diabetic neuropathy) reported substantial and sustained benefits of SCS at 12 months: mean pain reduction of 76% on VAS, improvements across neuropathic symptom scales (e.g., DN4 −49%, NPSI −73%). Importantly, distal intra-epidermal nerve fiber density (IENFD) increased by 22% in 12 months, suggesting potential small-fiber recovery. While limited by small sample size, single-center design, and mixed etiologies, these findings complement RCT data and support the plausibility of neurological improvement with SCS.

### 3.2. Patient Global Impression of Change (PGIC)

The PGIC was employed in several trials to capture the patient’s subjective perception of overall improvement, encompassing pain relief, functional capacity, and quality of life. At 6 months, Slangen et al. [24] reported that 59% of patients treated with SCS described a significant improvement in their condition according to PGIC scores, compared to only 7% in the conventional treatment group. Zuidema et al. [26] demonstrated the durability of this positive patient perception, with PGIC results remaining consistent over a 5-year follow-up. These findings closely mirrored the sustained pain reduction outcomes, further supporting the long-term clinical benefit of SCS in patients with PDN.

### 3.3. Quality of Life (EQ-5D, EQ VAS) and Quality-Adjusted Life Years (QALYs)

Several studies assessed the impact of SCS on the quality of life using validated tools such as the EQ-5D-5L, EQ Visual Analog Scale (EQ VAS), and estimations of quality-adjusted life years (QALYs). In the short term, the SENZA-PDN trial [38], Duarte et al. (2016) [28] reported significant improvements in both EQ-5D-5L index scores and EQ VAS after 6 months of treatment. Duarte et al. [28] specifically demonstrated that patients receiving SCS experienced greater gains in health-related quality of life compared to those treated with CMM. After adjusting for baseline differences, the SCS group showed a significantly higher increase in QALYs, indicating not only symptom relief but also enhanced overall health utility. At 24 months, Petersen et al. [25] confirmed the durability of these benefits, reporting sustained improvements in health-related quality of life (HRQoL) measures. In the long term, Yi Liu et al. (2025) [39] showed that EQ-5D-5L scores remained significantly elevated 5 years post-implantation. Remarkably, the average scores surpassed normative values for the general U.S. population aged 55–64 years, underscoring the lasting and meaningful impact of SCS on patients’ global health perception and daily functioning. Recently, Canós-Verdecho et al. [29] reported improvement HRQoL (EQ-VAS +69%; EQ-5D +134%).

### 3.4. Sleep Quality—Subjective, Short-, and Medium-Term

Improvement in sleep quality was a commonly reported secondary outcome, particularly assessed through patient-reported measures such as the PGIC. At 6 months, Slangen et al. reported that 36% of patients treated with SCS experienced noticeable improvements in sleep quality, whereas no improvement was observed in the control group receiving conventional medical therapy [24]. These benefits were not only immediate but also durable. Petersen et al. [25] documented sustained enhancements in sleep quality at 24 months, further supporting the long-term positive impact of SCS on sleep disturbances frequently associated with PDN. Also, the study of Canós-Verdecho et al. [29] confirmed improvements in sleep (−74%).

### 3.5. Neurological Function and Sensory Improvement—Objective, Short-, Medium-, and Long-Term

In addition to pain relief, several studies reported objective improvements in neurological function among patients treated with SCS. The SENZA-PDN trial demonstrated that SCS not only reduced pain intensity but also led to measurable improvements in neurological function, such as enhanced sensation and motor responses [38]. At 24 months, Petersen et al. confirmed that 65.7% of patients exhibited clinically meaningful neurological improvements, including recovery of sensory function. More recently, Yi Liu et al. [39] showed that these neurological benefits were sustained at 5 years, supporting the long-term therapeutic potential of 10 kHz SCS. Moreover, Zuidema et al. [26] and van Beek et al. employed the Michigan Diabetic Neuropathy Score (MDNS) to assess baseline neuropathy severity [40]. Their analyses revealed that patients with lower baseline MDNS experienced better outcomes, while those with more advanced neuropathy had a reduced likelihood of long-term improvement—highlighting the prognostic value of early intervention.

### 3.6. Peripheral Circulation (PtcO_2_, ABI, Vasodilation)—Objective, Short- and Medium-Term

In patients with ischemic diabetic foot, Zhou et al. [27] evaluated the effect of SCS on peripheral perfusion. At 6 and 12 months, SCS-treated patients showed significant increases in transcutaneous oxygen pressure (PtcO_2_) and ankle-brachial index (ABI), along with improved arterial vasodilation. These instrumental outcomes were associated with a reduced rate of amputations, suggesting that SCS may offer vascular benefits beyond neuropathic pain control.

### 3.7. Glucose Control Improvement—Objective, Short-, Medium-, and Long-Term

The resolution of chronic pain may positively influence glycemic control through mechanisms such as reduced stress response, improved sleep, and enhanced physical activity. However, despite these theoretical benefits, only two studies within the analyzed time frame have specifically evaluated and confirmed the impact of SCS on long-term glycemic control in patients with painful diabetic neuropathy.

An analysis of the Senza PDN study conducted by Klonoff et al. [36] demonstrated significant improvements in glucose control. After 2 years of treatment with SCS, patients with preoperative HbA1c >7% (53 mmol/mol) and >8% (64 mmol/mol) experienced statistically significant mean decreases of 0.5% (5.8 mmol/mol) and 1.1% (11.8 mmol/mol), respectively. This study highlights the potential of SCS to improve glycemic control, representing clinically meaningful improvements, but further research is needed to confirm these findings and explore longer-term outcomes.

In the case report described by Gazzeri et al., continuous glucose data monitoring reported mean 14-day glucose values of 222 ± 14 mg/dL and 172 ± 13 mg/dL, respectively, before and after DTM-SCS implantation [41]. The mean Area Under the Curve (AUC) values for hyperglycemia (glucose above 180 mg/dL) were 44 ± 13 mg/dl and 11 ± 6 mg/dL, respectively, before and after surgery. Furthermore, Time In Range (TIR) increased after neurostimulation from 36% to 52%, and Time Above Range (TAR) decreased after neurostimulation from 64% to 48%.

## 4. Economic Evaluation of SCS in PDN

An important consideration in the use of SCS for managing PDN is its cost-effectiveness relative to conventional medical treatment. Slangen et al. conducted a detailed economic evaluation alongside an RCT, comparing SCS combined with best medical treatment (BMT) versus BMT alone in 36 patients with refractory PDN [24]. At 12 months, the analysis showed that the total societal cost per patient was €26,539.18 in the SCS + BMT group compared to €5313.45 in the BMT-alone group. SCS was associated with higher gains in quality-adjusted life years (QALYs) as follows: 0.58 QALYs versus 0.36 QALYs, respectively. The resulting incremental cost-effectiveness ratio (ICER) was €94,159.56 per QALY gained and €34,518.85 per successfully treated patient. Bootstrap analyses revealed that the probability of SCS being cost-effective varied substantially—ranging from 0% to 46%—depending on the willingness-to-pay threshold (€20,000–€80,000 per QALY). Importantly, sensitivity analyses indicated that cost-effectiveness improved when baseline cost disparities were adjusted, when device costs were amortized over four years, and when clinical outcomes were projected over a longer time horizon.

## 5. Mechanisms of Action and Hypotheses on Spinal Cord Stimulation in Painful Diabetic Neuropathy

**Figure 2:**
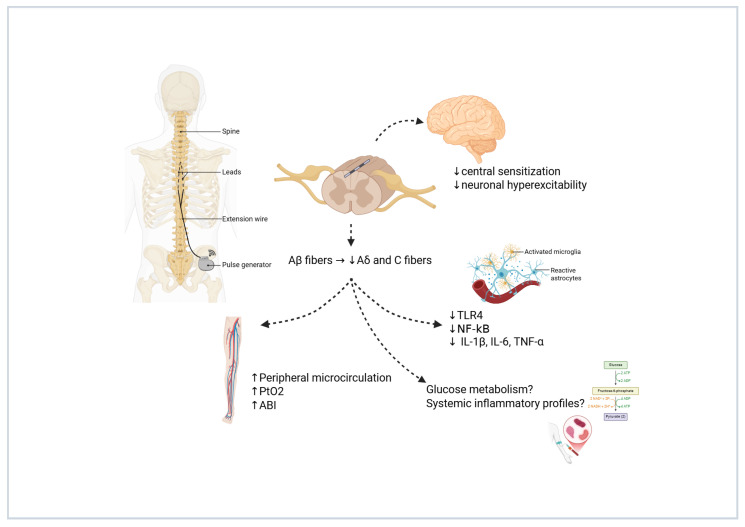
Multimodal mechanisms of action of SCS in PDN. This schematic illustrates the primary and secondary mechanisms underlying the analgesic and systemic effects of SCS. Activation of non-nociceptive Aβ fibers leads to inhibition of pain-transmitting Aδ and C fibers at the dorsal horn, resulting in reduced central sensitization and neuronal hyperexcitability. SCS also modulates neuroinflammation through downregulation of TLR4 and NF-κB signaling pathways, decreasing proinflammatory cytokines (IL-1β, IL-6, TNF-α). Additionally, improved peripheral microcirculation—evidenced by increased PtcO_2_ and ABI—may contribute to pain relief. Emerging hypotheses suggest SCS could influence systemic inflammatory profiles and glucose metabolism, highlighting its potential beyond localized neuromodulation. Created with BioRender.com. Varrassi, G. (2025). https://BioRender.com/83vra3a.

The precise mechanisms by which SCS alleviates pain in PDN are not yet fully understood; however, several converging hypotheses—derived from both clinical and preclinical studies—have been proposed. A foundational theory, supported by Strand et al. [42] and Raghu et al. [43], posits that SCS modulates dorsal horn neuronal activity by selectively inhibiting nociceptive fibers (Aδ and C fibers) while enhancing input from non-nociceptive Aβ fibers. This neuromodulatory effect likely contributes to the reduction in central sensitization and neuronal hyperexcitability—core features of chronic neuropathic pain [44]. Preclinical work by Wan Ni et al. [45], using a rat model of diabetic neuropathy, further elucidated the cellular and molecular pathways involved. Their study demonstrated that SCS suppresses neuroinflammation in the dorsal horn by downregulating Toll-like receptor 4 (TLR4) and nuclear factor-kappa B (NF-κB) signaling pathways. This led to reduced expression of proinflammatory cytokines, including interleukin-1β (IL-1β), interleukin-6 (IL-6), and tumor necrosis factor-alpha (TNF-α), indicating that modulation of the neuroimmune response plays a key role in the analgesic effect of SCS. Zhou et al. [27] contributed additional insight by emphasizing the vascular benefits of SCS in patients with ischemic diabetic foot ulcers. Their study showed that SCS improved peripheral perfusion metrics such as transcutaneous oxygen pressure (PtcO_2_) and ankle-brachial index (ABI). The resulting improvement in tissue oxygenation and reduction in ischemia may alleviate nociceptor sensitization, thereby reducing pain [46,47]. Furthermore, Yi Liu et al. [39] proposed a potential systemic component to SCS efficacy, observing that chronic neuromodulation might influence glucose metabolism and systemic inflammatory profiles in patients with type 2 diabetes and obesity. While the exact metabolic pathways remain to be clarified, these findings suggest that SCS may exert effects beyond the central and peripheral nervous system [48]. Overall, current evidence suggests that SCS alleviates PDN through a multimodal mechanism involving dorsal horn inhibition, reduction in central sensitization, suppression of neuroinflammation, improvement of peripheral microcirculation, and possible modulation of systemic metabolic and inflammatory pathways. Future research will be essential to delineate the relative contribution of each mechanism and to optimize stimulation parameters for maximal clinical benefit. Figure 2 summarizes the current understanding of the multimodal mechanisms by which SCS alleviates pain and improves physiological outcomes in patients with PDN.

## 6. Patient Selection and Exclusion Criteria for SCS in PDN

Patient selection for SCS in PDN has been consistent across major clinical trials, aiming to maximize therapeutic efficacy while minimizing procedural risks. Inclusion criteria across studies commonly required adult patients (≥18 years) with a confirmed diagnosis of type 1 or type 2 diabetes and chronic, bilateral neuropathic pain of at least 12 months’ duration, predominantly affecting the lower limbs [23,24,25,26]. Pain intensity had to be moderate to severe, typically quantified as a minimum score of 40–50 mm on a VAS or equivalent. Importantly, eligible patients were required to have failed to achieve adequate relief with optimized conventional medical therapy, including gabapentinoids, antidepressants, and—where appropriate—opioids, as per guideline-based treatment algorithms. Objective confirmation of PDN was often required, using clinical examination and/or validated scoring systems such as the MDNS [26,40]. A positive trial stimulation phase—commonly defined as achieving ≥50% pain reduction—was a mandatory prerequisite for permanent SCS implantation [23,24]. Exclusion criteria were similarly rigorous. Patients with poorly controlled diabetes (e.g., HbA1c > 10%), active infections, severe psychiatric disorders, cognitive impairment precluding accurate pain reporting, non-diabetic causes of neuropathy, or significant comorbidities increasing surgical risk (e.g., severe cardiopulmonary disease) were generally excluded [24,25,26,28]. In the SENZA-PDN study [25], additional exclusion criteria included mechanical instability of the spine and the inability to discontinue anticoagulant therapy due to increased procedural risk. Studies focused on specific diabetic complications, such as that by Zhou et al. [27] on ischemic diabetic foot ulcers, that added further vascular criteria—requiring evidence of critical limb ischemia while excluding patients with extensive tissue necrosis or active infection.

## 7. Proposed Clinical Decision Algorithm for SCS in Refractory PDN

Based on the converging evidence summarized above, we outline a pragmatic, evidence-informed algorithm to support referral, selection, and longitudinal management of patients with refractory PDN considered for SCS. This algorithm is intended as a clinician-facing aid, not a guideline, and should be adapted to local expertise, regulatory frameworks, and patient preferences.

### Proposed Clinical Algorithm for the Management of PDN

(1) Confirm the phenotype and optimize conventional care for a diagnostic confirmation of PDN. Ensure a typical distal symmetric neuropathic pain phenotype (burning, allodynia, paresthesia), supported, when feasible, by clinical scores and/or electrophysiology to exclude mimics (radiculopathy, vasculopathy, entrapment). Optimize CMM documenting a structured trial of first-line agents (e.g., duloxetine, pregabalin/gabapentin, tricyclics as appropriate) and adjuvants (topicals), including dose adequacy, adherence, and tolerability. Then, define refractoriness: persistent moderate–severe pain (e.g., NRS ≥ 6) and/or <50% improvement after adequate CMM (typically ≥8–12 weeks across ≥2 classes) constitutes failure of conservative therapy.
(2) Early referral and multidisciplinary evaluation. Consider SCS referral when (1) pain remains ≥6/10 or function is impaired; (2) sleep/HRQoL are significantly affected; (3) opioid escalation is anticipated or ongoing; (4) neuropathic exam suggests progressive sensory deficits despite CMM. Multidisciplinary review must be performed with neurosurgeon, pain specialist, diabetologist/endocrinologist, and where available a neuromodulation team review to check infection risk, glycemic control, ulceration/foot risk, anticoagulation status, psychological readiness, and realistic expectations (paresthesia-free vs. paresthesia-based paradigms, reprogramming needs).
(3) Pre-implant work-up and choice of implantation strategy. Pre-implant optimization may address modifiable risks (e.g., skin integrity, glycemic control as clinically appropriate) and provide antimicrobial/anticoagulation plans per local policy. Trial stimulation or direct implant must be addressed, selecting a pathway consistent with institutional practice and payer requirements. A short trial stimulation can support shared decision-making in ambiguous cases; single-stage implant may be preferred where trials are not mandated and infection/lead-migration risks weigh against an additional procedure.
(4) Implantation and initial programming. For lead placement and initial programming standardized targets and a documented programming protocol may be used. For paresthesia-free paradigms, verify comfort across common postures; for paresthesia-based paradigms, verify coverage of the painful area. Record initial parameters for reproducibility.
(5) Response evaluation and longitudinal follow-up. Core outcomes should be evaluated at baseline and at follow-up visits occurring approximately at 3, 6, 12, and 24 months (and beyond, where applicable). The assessed domains include the following:
  (a) Pain: responder rate (≥50% reduction from baseline) and mean change on NRS or VAS;
  (b) Patient-reported global impression of change (PGIC);
  (c) Function and interference (e.g., BPI-I), sleep quality, and health-related quality of life (HRQoL) (e.g., EQ-5D);
  (d) Neurological status (e.g., TCNS or MDNS, with optional QST or NCS when feasible);
  (e) Concomitant analgesic/opioid use and device-related safety outcomes (e.g., adverse events, lead migration, infection, explantation);
  (f) Metabolic and systemic parameters, including HbA1c and body weight/BMI, as exploratory markers of broader health impact.

Definition of success and durability. Primary success is defined with ≥50% pain relief and/or clinically meaningful improvement in HRQoL/function at 6–12 months, ideally sustained at 24 months. Durability (maintenance of responder status, time-to-loss of response) and reprogramming events may be reported.

(6) Reprogramming, rescue strategies, and alternative targets. Stepwise reprogramming should be undertaken when clinical response diminishes. Before labeling a therapy as ineffective, structured reprogramming—including parameter sweeps, waveform adjustments (e.g., tonic, burst, high-frequency), and spatial reconfiguration—should be systematically pursued. If inadequate response persists, lead revision or transition to dorsal root ganglion (DRG) stimulation may be appropriate, particularly in patients with focal or distal pain distributions. In selected phenotypes, combined or sequential stimulation strategies may serve as rescue options.

If SCS remains ineffective or complications arise, clinicians should revert to optimized CMM and consider enrollment in clinical trials investigating the following:

- Novel antidiabetic agents with potential neuroprotective effects (e.g., GLP-1 receptor agonists, SGLT2 inhibitors, dual incretin agonists);
- Next-generation neuropathic pain therapeutics, such as selective sodium channel blockers (NaV1.7, NaV1.8 inhibitors), anti-NGF antibodies, or gene- and RNA-based analgesic therapies;
- Innovative neuromodulation technologies, including closed-loop or adaptive stimulation platforms.
(7) Shared decision-making and documentation. Discuss expected benefits, uncertainties (e.g., long-term durability, device management), and lifestyle implications (charging/maintenance). Patient-level thresholds for success, follow-up schedule, and predefined actions for suboptimal response to minimize therapeutic drift should be recorded.

**Figure 3:**
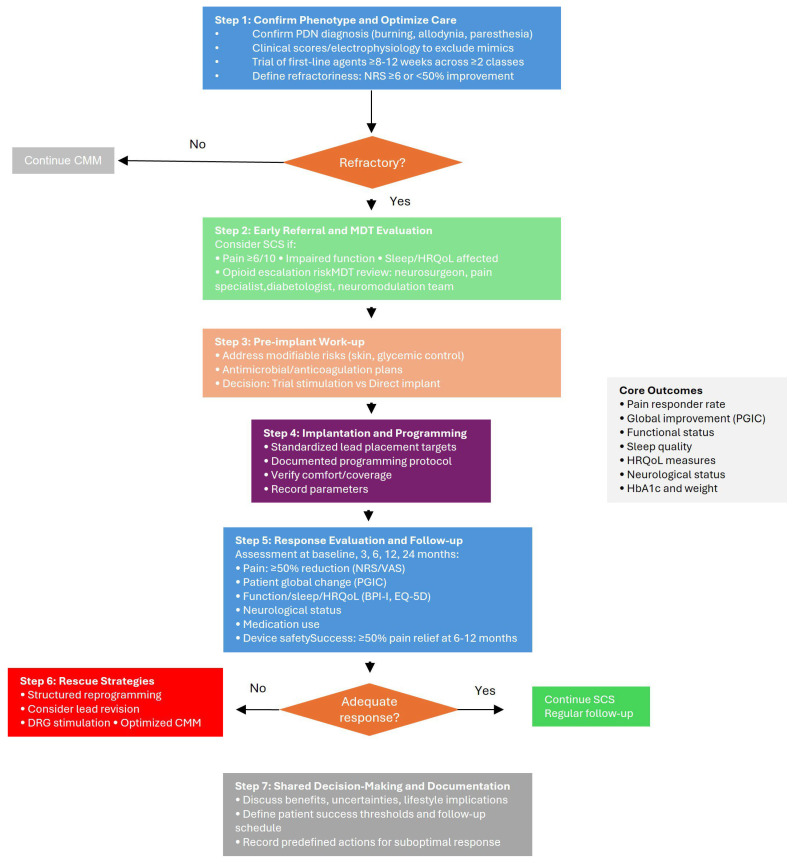
Proposed algorithm for referral, selection, and longitudinal management of SCS in refractory PDN.

This algorithm should be used to standardize reporting within and across centers and to align future studies on common outcome domains and timepoints (Figure 3).

## 8. Complications Associated with SCS: Incidence, Early and Late Events

The studies included in this review consistently reported that SCS for PDN is generally safe, although complications do occur, both early and late after implantation. Early complications were primarily related to surgical implantation procedures and included device-related infections, lead migration, hematoma formation, and wound healing issues. De Vos et al. (2014) [23] reported no major adverse events, although minor complications were not systematically detailed. Slangen et al. (2014) [24] documented one major adverse event—a fatal subdural hematoma—emphasizing the need for cautious patient selection, particularly regarding anticoagulation status. In the SENZA-PDN study [25], the infection rate requiring explantation was low (approximately 3.2%), and no stimulation-related neurological injuries were reported. Van Beek et al. (2018) [40] and Zuidema et al. (2023) [26] also described low infection rates, generally between 2% and 4%, primarily occurring within the first 6 months post-implantation. Traditionally, SCS implantation involves an initial trial phase to evaluate the patient’s response before proceeding with permanent device implantation [49]. However, emerging evidence has raised concerns that the trial phase, requiring two separate surgical procedures, may increase the risk of device-related infections [50]. Some recent studies suggest that direct implantation, without a prior trial, may be a viable option in selected patients, potentially reducing infection rates without compromising long-term clinical success [51,52,53]. The decision to include or omit the trial phase must be individualized, taking into account the patient’s clinical status, comorbidities, and overall risk profile. Moving forward, careful risk stratification and strict perioperative management will be crucial to minimizing infection risks while ensuring optimal therapeutic outcomes [54]. Bioabsorbable antibiotic-eluting envelopes (TyrX, Medtronic, Minneapolis, MN) may be used to reduce the risk of device infection. Used primarily in cardiac implantable electronic devices, these polypropylene envelopes release minocycline and rifampin for one week before the entire envelope is absorbed. An 87% decrease in infection in high-risk patients was reported with these antibiotic-eluting envelopes in the cardiac literature [55].

Minor early adverse events such as wound dehiscence and transient pain at the implantation site were self-limiting. Late complications were generally less frequent and included hardware-related failures (e.g., lead fracture, battery malfunction) and loss of stimulation efficacy over time. Zuidema et al. [26] reported that 80% of patients continued to use their SCS system at 5 years, suggesting relatively low long-term complication rates impacting device functionality. Van Beek et al. [40] similarly observed sustained device retention and function up to 5 years, although device replacement for battery depletion was occasionally necessary. Mechanical issues such as lead migration or breakage required surgical revision in a small proportion of cases (typically < 5%). Specific to patients with ischemic complications, Zhou et al. [27] did not report an increased risk of wound complications or ischemic deterioration related to SCS therapy, indicating that, with appropriate selection, even high-risk vascular patients can safely undergo SCS.

## 9. High Prevalence of PDN and Low Rates of SCS Implantation: Challenges and Strategies

Although the prevalence of PDN is substantial worldwide, the number of patients receiving SCS implants remains disproportionately low [56]. Several explanations have been proposed for this gap. A significant factor is the limited awareness among healthcare providers and patients regarding the proven benefits of SCS in PDN, leading to under-referral and under-utilization of this therapy [28]. In addition, concerns about the costs associated with SCS devices, despite evidence supporting their long-term cost-effectiveness, may discourage broader adoption [57]. Strict selection criteria for implantation, while intended to maximize safety and efficacy, might also unintentionally restrict access for many patients who could potentially benefit from neuromodulation. A multidisciplinary approach could be pivotal in addressing these challenges. Close collaboration between endocrinologists, diabetologists, neurologists, pain specialists, and neurosurgeons would facilitate earlier identification of appropriate candidates, optimize the management of comorbidities, and enhance patient education regarding the available therapeutic options. Multidisciplinary assessment not only ensures comprehensive evaluation but also improves long-term follow-up and adherence to therapy, thereby maximizing outcomes [58]. Looking ahead, several strategies could help improve the success rates and accessibility of SCS for patients with PDN. Enhancing educational initiatives targeted at both patients and healthcare providers, developing standardized clinical pathways for early identification and referral, and refining patient selection criteria based on predictive markers of response could collectively contribute to optimizing the use of SCS. Furthermore, ongoing clinical research focusing on personalized stimulation parameters and minimally invasive implantation techniques will be essential to expand the role of SCS in the management of PDN.

## 10. Future Perspectives in the Management of PDN with SCS

One major area of innovation in SCS for PDN is the development of new stimulation waveforms, not related to paresthesia elicitation. In recent years, four different SCS systems have been approved by the USA Food and Drug Administration (FDA) to treat PDN. The Nevro Corporation (Redwood City, CA, USA) was the first to announce on July 2021 approval of the Senza SCS system for management of chronic pain secondary to DPN. On January 2022 Medtronic (Minneapolis, MN, USA) announced approval by U.S. FDA of Intellis and Vanta (and later Inceptive) SCS systems for the treatment of PDN. On January 2023 Abbot (Plano, TX, USA) announced an expanded indication approval of Proclaim XR SCS system with Burst waveform to treat painful diabetic peripheral neuropathy. Finally, Boston Scientific (Valencia, CA, USA) was the last of the big SCS companies to win approval on October 2023, in the indication of the WaveWriter Alpha SCS for PDN treatment. In fact, high-frequency SCS, particularly the 10 kHz modality, has markedly changed clinical practice by offering effective pain relief without inducing paresthesia [31,59,60]. Traditional low-frequency stimulation requires the perception of paresthesia to be effective, which often leads to discomfort and reduced patient satisfaction [56,58]. High-frequency stimulation avoids this limitation and has shown superior outcomes in terms of sustained pain relief and improved patient-reported comfort. Similarly, low-frequency sub-threshold stimulation is also paresthesia-free, thus avoiding these discomfort-related limitations. Clinical studies have consistently supported the efficacy of 10 kHz SCS in delivering long-term analgesia, suggesting that future research should continue to explore and optimize these waveform technologies to further enhance therapeutic outcomes [25,61,62,63,64,65]. Yeung et al., in their recent review of SCS for PDN, concluded that high frequency 10 kHz SCS, compared with traditional SCS, offered significant pain reduction and improved quality of life by eliminating paresthesia [64]. Recently, Differential Target Multiplexed (DTM) SCS showed interesting results in the treatment of PDN [41]. DTM-SCS is a novel paresthesia-free stimulation technique targeting the supportive glial cells in the nervous system, modulating glial cells and neurons with a rebalance of their interactions and represents an effective tool with a symptomatic role in reduction in painful symptoms [57], but also a therapeutic role in the reduction in glucose levels [41]. Although DTM-SCS showed effectiveness in decreasing the average glucose level with a reduction in insulin requirement in a PDN patient [41], these results should be examined and ideally validated in a larger independent cohort of patients to ensure their effectiveness.

Closed loop SCS represents a transformative development in neuromodulation techniques, offering adaptive pain management that differs from traditional SCS systems, which deliver stimuli to the spinal cord with fixed output [66]. Closed loop SCS is a novel advanced technology that provides more focused stimulation, adapting to the movement and physiological activity of the spinal cord through continuous evoked compound action potential (ECAP) measurement and subsequent amplitude adjustment. Although emerging studies suggest closed loop SCS potential for pathologic conditions such as neuropathic pain, no studies have been published on PDN.

The integration of artificial intelligence (AI) and machine learning models into clinical practice holds great promise for enhancing patient selection and optimizing outcomes of SCS in PDN, by enabling the development of predictive algorithms based on multidimensional clinical, neurophysiological, and imaging data [67]. However, dedicated studies specifically exploring the application of AI and machine learning in predicting SCS outcomes for PDN are urgently needed to validate this promising approach and clarify its clinical utility.

### Dorsal Root Ganglion (DRG) Involvement in PDN: From Pathogenesis to Neuromodulation

**Figure 4:**
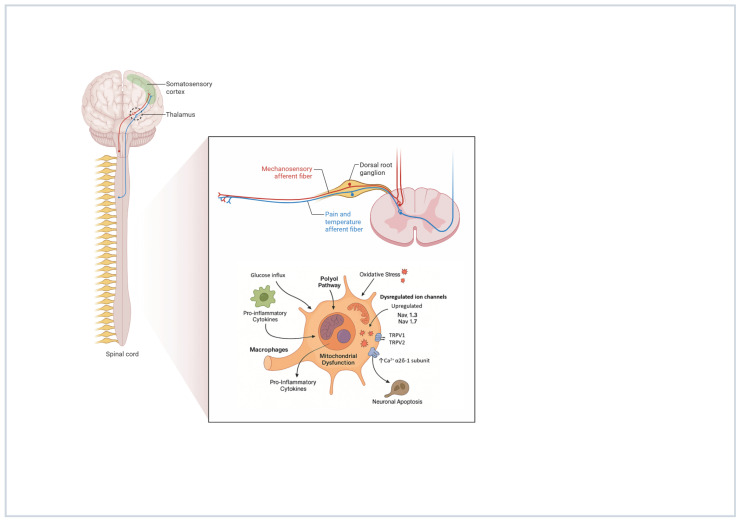
Pathological alterations occurring in the DRG during PDN. Created with BioRender.com. Varrassi, G. (2025). https://BioRender.com/i3ipkhm.

The DRG plays a pivotal role in the pathophysiology of PDN, serving as a critical site of neuronal damage and dysfunction. The DRG contains the cell bodies of primary sensory neurons and lacks a blood–nerve barrier, making it particularly vulnerable to hyperglycemia-induced metabolic insults and oxidative stress [68]. Chronic hyperglycemia triggers multiple pathological mechanisms within DRG neurons, including increased polyol pathway flux, advanced glycation end-product formation, protein kinase C activation, and enhanced oxidative stress, leading to mitochondrial dysfunction and neuronal apoptosis [69]. In fact, DRG neurons exhibit early morphological changes in PDN, including neuronal swelling, mitochondrial abnormalities, and altered gene expression profiles affecting neurotrophic support and ion channel function [70]. Furthermore, inflammation within the DRG, characterized by macrophage infiltration and proinflammatory cytokine production, contributes to the development and maintenance of neuropathic pain in diabetic patients, suggesting that the DRG represents not only a site of neuronal injury but also a potential therapeutic target for managing PDN [3]. Within the DRG, voltage-gated sodium channels undergo significant dysregulation during PDN leading to elevated expression of specific channel subtypes including Nav1.3 and Nav1.7 in animal models [71,72]. These sensory neurons experience prolonged sodium channel activation periods, disrupting normal neuronal excitability patterns and contributing to hyperglycemia-induced impairment of cellular ion transport mechanisms [73]. The DRG also contains co-localized pain-sensing receptors TRPV1 and TRPA1 within specific neuronal populations, which become activated by inflammatory mediators and reactive oxygen species, ultimately generating abnormal pain signaling [74]. Additionally, calcium channel dysfunction occurs through overexpression of the α2δ-1 subunit, amplifying intracellular calcium influx and triggering downstream cascades involving protein kinase C phosphorylation and TRPV upregulation [75,76]. These interconnected pathological changes within DRG neurons contribute to the complex pathophysiology of PDN through multiple converging mechanisms involving ion channel dysfunction, inflammatory signaling, and cellular stress responses [77]. In Figure 4, we illustrate the pathological alterations occurring in the DRG during PDN, highlighting key neuronal and molecular mechanisms. Several of these disrupted pathways may serve as potential therapeutic targets for DRG neuromodulation.

DRG has emerged as a promising and innovative target in the field of neuromodulation. Unlike traditional SCS, DRG stimulation can engage not only Aβ-fibers but also C- and Aδ-fibers, owing to the distinct anatomical and physiological characteristics of the DRG [78]. This technique has garnered substantial scientific support, particularly for its efficacy in treating complex regional pain syndrome (CRPS) [79]. In the context of PDN, the DRG represents a promising target. In fact, a recent clinical investigation demonstrated that electrical stimulation of DRG offers promising results in the treatment of refractory PDN, with patients receiving permanent implants experiencing an average pain reduction of over 60% [80]. Moreover, an experimental study using DRG stimulation in a PDN animal model has shown significant pain reduction without corresponding changes in intracellular GABA immunofluorescence intensity within DRG sensory neurons, suggesting that pain relief mechanisms may involve pathways beyond GABAergic signaling modulation [81]. Similarly, the same group published a comparative experimental study demonstrating that both conventional and burst DRG stimulation effectively normalize mechanical hypersensitivity in animal models of PDN. Notably, DRG burst stimulation appears to offer potential advantages by providing sustained residual analgesic effects even after the cessation of electrical stimulation [82]. Finally, a prospective cohort study comparing SCS and DRG stimulation in 106 patients with PDN demonstrated comparable therapeutic efficacy between both neuromodulation approaches, with approximately 80% of patients achieving clinically significant pain reduction and similar improvements in quality-of-life measures at 12-month follow-up [83]. Although preliminary clinical and preclinical findings are promising, further well-designed, large-scale, and long-term studies are needed to draw definitive conclusions and establish clear clinical indications for the use of DRG neurostimulation in the treatment of PDN.

## 11. Discussion and Conclusions

SCS has emerged as a promising therapeutic option in the management of PDN, particularly in patients with chronic, refractory symptoms unresponsive to conventional pharmacological therapy. This review synthesizes evidence from RCTs and long-term observational studies to provide a comprehensive assessment of the safety, efficacy, and mechanisms of action of SCS in this context. SCS consistently achieved ≥50% pain reduction in the majority of patients, with durability extending up to five years. Additionally, improvements were noted in the quality of life, sleep, neurological function, and patient-reported outcomes. Importantly, SCS demonstrated a favorable safety profile, with a low incidence of serious complications. Most adverse events were minor or device-related. The integration of validated tools such as the PGIC, EQ-5D, and MDNS across studies reinforces the robustness of these findings. Mechanistically, SCS appears to exert multifactorial effects. It modulates dorsal horn activity, inhibits nociceptive transmission, reduces neuroinflammation via TLR4 and NF-κB pathways, and enhances peripheral microcirculation. Moreover, early evidence suggests potential systemic metabolic effects, possibly influencing glucose homeostasis, though this remains an area for future investigation. Despite compelling clinical data, SCS remains underutilized in PDN management. Barriers include limited awareness, perceived costs, and stringent selection criteria [37]. Addressing these issues through multidisciplinary collaboration and education is essential to improving access and optimizing outcomes.

Moreover, this review provides distinctly novel clinical insights into two emerging areas of SCS application in PDN. First, we highlight the potential role of DRG modulation as a mechanistic target, underscoring how neurostimulation may influence neuroinflammatory processes at the DRG level—an aspect rarely addressed in previous reviews. Second, we discuss preliminary evidence suggesting that SCS may also affect glucose metabolism, representing a potential paradigm shift from viewing SCS solely as an analgesic intervention to considering its broader systemic metabolic implications. These dual perspectives—DRG-targeted mechanisms and glucose regulation—not only expand the conventional framework of SCS in PDN but also distinguish this review by linking neurostimulation techniques with emerging metabolic outcomes in diabetic patients. Although current data are limited, various studies support the efficacy and safety of SCS—particularly new waveforms paradigms—for the treatment of PDN refractory to medical management. Given its effects on pain relief, functional improvement, and quality of life, SCS should be considered an option within the treatment algorithm for carefully selected patients with severe, refractory PDN. The inclusion of studies from 2014 reveals a concerning pattern: despite nearly a decade of research, significant heterogeneity persists in stimulation parameters, outcome measures, and evaluation timepoints. This variability across both early and recent studies underscores the urgent need for standardized, high-quality trials with consistent protocols to establish definitive evidence for SCS efficacy in PDN. Furthermore, we believe that future studies may highlight the efficacy of SCS also on glycometabolic control, glucose variability, and hyperglycemic risk.

## Figures and Tables

**Table 1:** Summary of clinical studies on SCS in PDN.

Author, Year	Journal	Study Design	Sample Size	Primary Outcome	SCS Parameters	Overall Risk of Bias
De Vos et al. 2014 [23]	Pain	Multicenter randomized clinical trial	60 patients	**✓** Proportion of patients with 50% pain reduction Primary outcome obtained at 6 months follow-up	**✗** Stimulation parameters not reported.	**✗** High (Lack of blinding for a subjective pain outcome; allocation concealment not clearly reported)
Slangen et al. 2014 [24]	Diabetes Care	Prospective two-center randomized controlled trial	36 patients	**✓** Proportion of patients with 50% pain reduction 59% of SCS patients obtained primary outcome at 6 months follow-up	**✗** Stimulation parameters not reported.	**✗** High (Open-label design with subjective pain outcome and small sample size)
Petersen et al. 2021 [25]	JAMA Neurology	Randomized clinical trial	216 patients	**✓** 50% pain reduction and no deterioration on neurological examination 79% of SCS patients obtained primary outcome at 6 months follow-up	**✓** 10 kHz frequency, 30 μs pulse width delivered via bipole, amplitude range of 0.5 to 3.5 mA	**✗** High (Pain outcome measured without participant/outcome-assessor blinding, despite otherwise strong methodology)
Zuidema et al. 2023 [26]	Neuromodulation	Prospective cohort study	19 patients	**✓** Pain intensity reduction (day and night) >50% of patients, the pain reduction was >30% at eight-to-ten-year follow-up	**✓** Bipool configuration, pulse width 150–450 μm. Fewer differences were present in stimulation frequency, with most (65%) patients frequency of 30 Hz, although higher frequencies up to 60 Hz were also used.	**✗** Critical (Uncontrolled confounding; no randomized comparator)
Zhou et al. 2024 [27]	Neurosurgery	Retrospective cohort study	141 patients	**✓** Comparison of amputation rates between SCS and TDC groups. Odds of amputation at 12 months: OR = 0.17 (95% CI, 0.08–0.37)	**✓** Voltage, 0.5 V; pulse width, 180–240 μs; frequency, 40 Hz	**◐** Serious (Treatment allocation based on patient preference; limited statistical adjustment)
Duarte et al. 2016 [28]	Quality of Life Research	Multicenter randomized controlled trial	60 patients (CMP = 20, SCS = 40)	**✓** SCS vs. CMP at 6 months (QALY gain) QALY gain—adjusted for baseline EQ-5D score = 0.258	**✗** Stimulation parameters not reported.	**✗** High (Shares the parent trial’s lack of blinding; quality-of-life outcome self-reported)
Canós-Verdecho et al. 2025 [29]	Journal Clinical Medicine	Prospective observational cohort study	20 patients (6DPN)	**◐** Pain intensity reduction and potential small fiber re-growth	**✓** Combination of paresthesia-based stimulation and Contour© (50 Hz, ~300 μs pulse-width, ~40% of perception threshold), or FAST (90 Hz frequency, ~250 μs pulse-wid, 40% of perception threshold)	**◐** Serious (non-randomized; small N; mixed etiologies)

Legend: **✓** is the target performance, **✗** is substandard performance, and **◐** is marginal performance.

**Table 2:** Core outcomes across the SENZA-PDN randomized program and extensions. Overview of responder rates (≥50% pain relief), mean pain change, neurological improvement, HRQoL (EQ-5D/DQOL), sleep interference, patient satisfaction, and safety at 6, 12, 24 months, and ~4 years.

Author, Year	Journal	Study Type	Dataset Phase	Sample Size	Follow Up	Key Outcomes
Mekhail, 2020 [30]	Trials	Trial protocol/design (SENZA-PDN RCT)	Design	Planned RCT; design paper	Describes 3–6 months primary, longer-term extensions	Protocol for RCT comparing 10 kHz SCS + CMM vs. CMM in refractory PDN
Petersen, 2021 [25]	JAMA Neurology	Randomized clinical trial (primary outcomes)	Randomization	216 randomized; 187 assessed at 6 months	Primary endpoint at 3 months; 6-months randomized phase	≥50% pain relief: 85% SCS vs. 5% CMM at 6 months; HRQoL and sleep improved; acceptable safety
Petersen, 2022 [31]	Diabetes Care	RCT follow-up	12 months	Original SCS: 84; CMM → SCS crossover: 58	12 months	Durable pain relief; high responder rates; neurological improvement persists; crossover similar after implant
Petersen, 2022 [32]	Mayo Clin Proc Innov Qual Outcomes	RCT outcomes analysis (patient-centered outcomes)	12 months	RCT cohort	12 months	HRQoL (EQ-5D) and satisfaction improved alongside large pain reductions
Petersen, 2023 [33]	Diabetes Research and Clinical Practice	RCT extended outcomes	24 months	142 with SCS (84 initial + 58 crossover)	24 months	Mean pain −79.9%; 90.1% ≥50% relief; 65.7% neurological improvement; HRQoL and sleep improved; 3.2% explants (infection)
Taylor, 2023 [34]	J Manag Care Spec Pharm	Health economics/utilization (RCT)	0–6 months randomized phase	RCT resource-use dataset	6 months (annualized costs)	Lower hospitalizations and total healthcare costs with 10 kHz SCS + CMM vs. CMM
Argoff, 2025 [35]	J Diabetes Sci Technol	Subanalysis: protective sensation/ulceration risk	3-, 6-, 12-, 24-months assessments	RCT cohort incl. crossover	Up to 24 months	More sensate monofilament sites; low-risk ulceration class roughly doubled by 3 months and sustained to 24 months
Klonoff, 2024 [36]	Journal of Pain Research	Post hoc subanalysis: metabolic and sleep outcomes	24 months	SENZA-PDN participants with T2D	24 months	HbA1c and body weight reduced (largest in higher baseline HbA1c/BMI); sleep interference reduced
Petersen, 2025 [37]	Pain Practice	Post-study survey (real-world, long-term)	≈4.1 years post-implant	Implanted SENZA-PDN patients (survey)	≈4.1 years	Sustained pain relief and HRQoL; no explants for loss of efficacy; weight and HbA1c reductions vs. 24 months

## Data Availability

No new data were created or analyzed in this study. Data sharing is not applicable to this article.

## Abbreviations

The following abbreviations are used in this manuscript:

ABI	Ankle-Brachial Index
AI	Artificial Intelligence
ALA	Alpha-Lipoic Acid
ALC	Acetyl-L-Carnitine
BMT	Best Medical Treatment
CMM	Conventional Medical Management
CMP	Conventional Medical Practice
CMT	Conventional Medical Therapy
DTM	Differential Target Multiplexed
DN	Diabetic Neuropathy
EQ-5D-5L	EuroQol 5-Dimension 5-Level Scale
EQ VAS	EuroQol Visual Analog Scale
GLA	Gamma-Linolenic Acid
HRQoL	Health-Related Quality of Life
ICER	Incremental Cost-Effectiveness Ratio
IL-1β	Interleukin-1 beta
IL-6	Interleukin-6
MDNS	Michigan Diabetic Neuropathy Score
ML	Machine Learning
NNH	Number Needed to Harm
NNT	Number Needed to Treat
NF-κB	Nuclear Factor Kappa-light-chain-enhancer of Activated B cells
NRS	Numeric Rating Scale
PEA	Palmitoylethanolamide
PDN	Painful Diabetic Neuropathy
PGIC	Patient Global Impression of Change
PtcO_2_	Transcutaneous Oxygen Pressure
QALY	Quality-Adjusted Life Year
RCT	Randomized Controlled Trial
SCS	Spinal Cord Stimulation
SNRI	Serotonin-Norepinephrine Reuptake Inhibitor
TDC	Traditional Debridement Care
TLR4	Toll-like Receptor 4
TNF-α	Tumor Necrosis Factor-alpha
VAS	Visual Analog Scale
